# Endothelial progenitor cells transplantation attenuated blood-brain barrier damage after ischemia in diabetic mice via HIF-1α

**DOI:** 10.1186/s13287-017-0605-3

**Published:** 2017-07-11

**Authors:** Jieli Geng, Liping Wang, Meijie Qu, Yaying Song, Xiaojie Lin, Yajing Chen, Muyassar Mamtilahun, Shengdi Chen, Zhijun Zhang, Yongting Wang, Guo-Yuan Yang

**Affiliations:** 1 Department of Neurology, Shanghai Ruijin Hospital, School of Medicine, Shanghai Jiao Tong University, Shanghai, 200025 China; 2 Department of Neurology, Shanghai Renji Hospital, School of Medicine, Shanghai Jiao Tong University, Shanghai, 200127 China; 3 Neuroscience and Neuroengineering Research Center, Med-X Research Institute and School of Biomedical Engineering, Shanghai Jiao Tong University, 1954 Hua Shan Road, Shanghai, 200030 China

**Keywords:** Blood-brain barrier, Diabetes, Endothelial progenitor cell, HIF-1α, Ischemic stroke

## Abstract

**Background:**

Blood-brain barrier impairment is a major indicator of endothelial dysfunction in diabetes. Studies showed that endothelial progenitor cell (EPC) transplantation promoted angiogenesis and improved function recovery after hind limb ischemia in diabetic mice. The effect of EPC transplantation on blood-brain barrier integrity after cerebral ischemia in diabetic animals is unknown. The aim of this study is to explore the effect of EPC transplantation on the integrity of the blood-brain barrier after cerebral ischemia in diabetic mice.

**Methods:**

EPCs were isolated by density gradient centrifugation and characterized by flow cytometry and immunostaining. Diabetes was induced in adult male C57BL/6 mice by a single injection of streptozotocin at 4 weeks before surgery. Diabetic mice underwent 90-minute transient middle cerebral artery occlusion surgery and received 1 × 10^6^ EPCs transplantation immediately after reperfusion. Brain infarct volume, blood-brain barrier permeability, tight junction protein expression, and hypoxia inducible factor-1α (HIF-1α) mRNA level were examined after treatment.

**Results:**

We demonstrated that neurological deficits were attenuated and brain infarct volume was reduced in EPC-transplanted diabetic mice after transient cerebral ischemia compared to the controls (*p* < 0.05). Blood-brain barrier leakage and tight junction protein degradation were reduced in EPC-transplanted mice (*p* <0.05). EPCs upregulated HIF-1α expression while HIF-1α inhibitor PX-478 abolished the beneficial effect of EPCs.

**Conclusions:**

We conclude that EPCs protected blood-brain barrier integrity after focal ischemia in diabetic mice through upregulation of HIF-1α signaling.

## Background

Diabetes is an independent risk factor for ischemic stroke [1]. It also increases the risk of disability and death after ischemic stroke [2]. The progressive dysfunction of the endothelium in diabetes is related to the poor prognosis. A recent clinical study reported that acute ischemic stroke patients with diabetes have a higher risk for symptomatic intracerebral hemorrhage following thrombolytic therapy compared to non-diabetic stroke patients [3]. It was also observed that tissue plasminogen activator (tPA) treatment increased blood-brain barrier (BBB) leakage and brain hemorrhage, which may be due to the increase of inflammatory response after embolic middle cerebral artery occlusion (MCAO) in type-1 diabetic rats [4]. BBB disruption and hemorrhagic transformation increases ischemic lesion and deteriorates neurological function.

Stem cell therapy holds great promises to treat ischemic stroke [5–7]. Mesenchymal stem cell (MSC) transplantation improved functional recovery after ischemic stroke in clinical trials in humans [8]. Beneficial effect of MSC transplantation was observed after focal ischemia in non-diabetic rats but not in diabetic rats [9]. Studies showed that MSC therapy for ischemic stroke in diabetic rats increased BBB leakage and brain hemorrhage, which could counteract the beneficial effect of MSCs [10]. Recent studies also showed that endothelial progenitor cell (EPC) administration could enhance angiogenesis and improve neurological function during the post-acute phase of ischemic stroke [11–13]. However, the effect of EPCs on the endothelial repair and neurological functional recovery in diabetes after ischemic stroke is unclear. Since BBB impairment is a part of both the microangiopathy in diabetes and the brain injury in the acute phase of ischemic stroke, we therefore chose the BBB damage during the acute phase of ischemic stroke as our research subject.

Endothelial cells of microvessels are the main component of the BBB in the brain. The derangement of endothelial structure and function is an essential pathological mechanism in diabetic microangiopathy [14]. Studies in vitro and in vivo showed that microvascular disorder caused by diabetes could lead to the impairment of BBB integrity and increase BBB permeability [15–17]. However, numerous studies about diabetes-induced microangiopathy were conducted outside the brain [18–21], which did not mimic structural changes of BBB in diabetic mice [22]. Studies demonstrated that stem cell therapy had a great potential for the attenuation of microangiopathy in diabetic animal models [23] including diabetic retinopathy [24], diabetic nephropathy [25], diabetic cardiomyopathy [26, 27], and diabetic neuropathy [28–30]. The beneficial effects of stem cell therapy include pro-angiogenesis [31], neurotropy [32], anti-apoptosis [33], anti-inflammation [25], anti-fibrosis [34], and anti-oxidative stress [35, 36]. Moreover, EPC is a promising candidate of stem cell therapy for the attenuation of microangiopathy caused by diabetes since EPC exhibited more efficacy of protection against oxidative stress than mature endothelial cells [37].

Hypoxia-inducible factor-1α (HIF-1α) is a critical transcription factor in maintaining oxygen homeostasis in physiological conditions and in regulating the cellular adaptive reaction under hypoxic conditions [38]. Its target genes are involved in many important processes such as angiogenesis, cell proliferation and energy metabolism [39]. HIF-1α is a major genetic modifier in coronary artery disease [40, 41]; and it protects the heart against ischemic-reperfusion injury in mice [42]. HIF-1α also showed neuroprotective effect in ischemic stroke [43–46]. A recent study reported that transplantation of EPCs restored the local blood flow and improved limb function after unilateral hind limb ischemia in diabetic mice by inducing HIF-1α hyperexpression [47]. However, whether the HIF-1α pathway is involved in the mechanisms of EPC therapy in treating ischemic brain injury in diabetes remains unclear.

In this study, we investigated whether EPC transplantation could rescue ischemic brain injury by attenuating BBB disruption during the acute phase of ischemic stroke in diabetic mice. And if so, whether the HIF-1α pathway is involved in the beneficial effect of EPCs.

## Methods

### Experimental design

Animal experimental procedures were approved by the Institutional Animal Care and Use Committee of Shanghai Jiao Tong University, Shanghai, China. Adult male C57BL/6 mice (n = 145) weighing 20–25 grams were housed with free access to food and water under a 12 h light-dark cycle (light on at 8:00, light off at 20:00) for 1–2 weeks prior to the experiment. Diabetes mellitus was induced by a single injection of streptozotocin (STZ). The experiment contained two parts. In part one, in order to investigate the effect of EPC therapy on the brain injury after MCAO, the diabetic mice were randomly divided into three groups: an EPC-treated group, phosphate-buffered saline (PBS)-treated group and sham group. In part two, we investigated the role of HIF-1α in EPC-treated diabetic mice after tMCAO, and the diabetic mice were divided into PBS-treated, EPC-treated, HIF-1α inhibitor PX-478-treated and EPC plus PX-478 co-treated groups.

### EPC isolation from human umbilical cord blood

EPC was isolated from human umbilical cord blood obtained from International Peace Maternity and Child Health Hospital, Shanghai, China. This procedure was approved by the Ethics Committee of Shanghai Jiao Tong University, Shanghai, China. EPCs were isolated as previously described [48]. Briefly, the blood was diluted by sterile PBS at 1:1 and then layered carefully on top of lymphocyte separation medium (MP Biomedicals, Santa Ana, CA, USA) at 4:3 v/v. Subsequently, mononuclear cells were isolated by density gradient centrifugation for 30 min at 400 *g* at 4 °C. Buffy coat mononuclear cells were transferred to a new sterile conical tube. Cells were then washed twice with M199 medium and centrifuged at 200 *g* at 4 °C to remove the lymphocyte separation medium. After final centrifugation, supernatant was carefully discarded and cellular pellet was re-suspended in EGM-2 Bullet Kit (Lonza, Walkersville, MD, USA), which is consisted of 2% fetal bovine serum, rhEGF, VEGF, rhFGF-B, R3-IGF-1, GA-1000, ascorbic acid, hydrocortisone and heparin. The cell suspension was transferred to a six-well plate coated with collagen type I (Corning, Tewksbury, MA, USA) in advance, and incubated at 37 °C with 5% CO_2_. The culture medium was partly replaced by fresh EGM-2 after 48 h of cell seeding and then every 3 days. After 28 days of culture, expended EPCs reached 90% confluence and was trypsinized and passaged. The EPCs cultured within 6–8 passages were used for this study.

### Characterization of EPCs

EPCs were characterized by flow cytometry analysis and immunofluorescent staining. For flow cytometry, EPCs were incubated with fluorescent-labeled mouse anti-human antibodies against CD34 (eBioscience, San Diego, CA, USA), CD133 (Miltenyi Biotec, Auburn, CA, USA), CD31 (eBioscience), KDR (BD Biosciences, Franklin Lakes, NJ, USA), CD45, CD29 and CD90 (BD Biosciences) for 30 min at room temperature. Cells were analyzed by a flow cytometry (BD Biosciences) after washing and re-suspension with PBS. For immunofluorescent staining, glass slides with EPCs were fixed with 4% formaldehyde for 10 min and blocked by bovine serum albumin for 1 h at room temperature. Then slides with EPC cells were incubated with primary antibodies against CD34 (1:50, R&D Systems, Minneapolis, MN, USA), KDR (1:10, R&D Systems), CD31 (1:20, R&D Systems) and vWF (1:400, Abcam, Cambridge, MA, USA) overnight at 4 °C, followed by PBS rinse three times and incubation with secondary antibodies for 1 h at room temperature. Finally, the cells were examined under a laser scanning confocal microscope (Leica, Solms, Germany).

### Induction of mouse diabetes mellitus model

Diabetes was induced by a single injection of STZ (150 mg/kg, Sigma-Aldrich, St. Louis, MO, USA). STZ was freshly dissolved in 0.1 M sodium citrate buffer (pH 4.5) to a final concentration of 10 mg/ml and used within 20 min. Before STZ administration, mice were fasted (water was available) for 10 h. After STZ injection, mice were allowed access to food and water freely. At 7 days after STZ injection, diabetes status was assessed by measuring serum glucose levels using the glucose oxidase method (Bayer HealthCare LLc, Mishawaka, IN, USA). Diabetes is defined as serum glucose concentration being above 300 mg/dl. The serum glucose level was checked again at the day of tMCAO procedure to confirm hyperglycemia.

### Transient MCAO in mice

After 4 weeks of STZ injection, the 90-min transient MCAO (tMCAO) model was established. Transient MCAO was performed as previous described [49]. Briefly, mice were placed in the supine position after anesthesia with ketamine (100 mg/kg, Fujian Gutian Pharmaceutical Co., Ltd, Gutian, China)/xylazine (10 mg/kg, Sigma-Aldrich). After the midline incision was made on the neck, a silicone-coated 6-0 suture (Covidien, Mansfield, MA, USA) was inserted from a small incision of the left external carotid artery and gently advanced into internal carotid artery to occlude the origin of middle cerebral artery. The total distance from the external carotid artery to middle cerebral artery was approximate 9 ± 0.5 mm. Reperfusion was achieved by withdrawing the suture after 90 min of tMCAO. Both occlusion and reperfusion were confirmed by laser Doppler flowmetry (Moor Instruments, Axminster, UK). The filament was removed immediately after insertion in the middle cerebral artery in the sham mice.

### Administration of EPCs and PX-478

Animals were randomly divided into four groups designated as PBS, EPC, EPC plus PX-478-treated and PX-478-treated groups. 1 × 10^6^ EPCs suspended in 100 μl PBS was administered at the speed of 20 μl per minute through the left jugular vein immediately after reperfusion. The same amount of PBS was administrated as a control. PX-478 (40 mg/kg, Selleck, Houston, TX, USA) was injected intraperitoneally immediately after EPC administration.

### Assessment of neurological severity score

At 24 h after tMCAO, modified neurological severity score (mNSS) was used to evaluate the neurological function by an investigator who was blind to the treatment design. The mNSS scoring evaluate motor, sensory, balance and reflex functions and is expressed as a range from 0 to 14, as reported [49].

### Measurement of brain infarct volume

For brain infarct volume measurement, a series of 20 μm coronal sections were cut from the anterior commissure to hippocampus with 200 μm interval. The sections were stained with Cresyl Violet (Sigma-Aldrich). NIH ImageJ software (National Institutes of Health, Bethesda, MD, USA) was used to delineate the infarct area. Infarct volume was calculated as described previously [50].

### Immunohistochemistry

BBB permeability was assessed by measuring the extravasation of immunoglobulin G (IgG). As previously described [49], Vectastain Universal ABC Kit (Vector Laboratories, Burlingame, CA, USA) was used for IgG staining. Briefly, brain slices were fixed with 4% paraformaldehyde and blocked with BSA. Slices were then incubated with biotinylated secondary antibody and ABC reagent (Vector Laboratories) for 30 min, followed by incubation with DAB reagent (Vector Laboratories) and counterstained with hematoxylin. Images were collected in three random fields along the ischemic penumbra and analyzed with IPP software (Image Pro Plus 6.0, Media Cybernetics, Bethesda, MD, USA). For immunostaining of CD31, occludin, ZO-1 and claudin-5 and HIF-1α brain slices were fixed with methanol at room temperature for 20 min and blocked with diluted donkey serum (Jackson ImmunoResearch, West Grove, PA, USA) for 60 min at room temperature. Slides were incubated overnight at 4 °C with primary antibodies of CD31 (1:500, R&D Systems), occludin (1:200, Life Technologies, Carlsbad, CA, USA), zonula occludens-1 (ZO-1, 1:100, Life Technologies), claudin-5 (1:200, Life Technologies), HIF-1α (1:50, Proteintech, Wuhan, China). After rinsing with PBS, brain sections were incubated with the fluorescence-conjugated second antibodies for 1 h at room temperature. Brain sections were photographed using a confocal microscope (Leica). At least four vessels were randomly chosen in the perifocal region per brain section, and total eight sections were selected per animal. The length of vessels and gap were quantified by ImageJ software (National Institutes of Health). Gap length was presented as percentage (%) of whole tight junction (TJ) staining as previously reported [49].

### Western blot analysis

Regional brain tissue including the ischemic core and penumbra was sectioned for Western blotting analysis, and the corresponding region from the contralateral hemisphere was used as a control. Protein samples extracted from brain sections were denatured by for 10 min at 95 °C. Forty micrograms denatured protein was subjected to SDS-PAGE electrophoresis on 10% gel, then electroblotted to nitrocellulose membrane at 300 mA for 90 min. The membrane was then incubated with the following primary antibodies overnight at 4 °C: occludin (1:1000, Life Technologies), ZO-1 (1:1000, Life Technologies) and β-actin (1:1000, Santa Cruz Biotechnology, Dallas, TX, USA). After washing, the membrane was incubated with horseradish peroxidase-conjugated secondary antibodies for 1 h at room temperature and visualized by chemiluminescent (Pierce, Rockford, IL, USA). The final bands were visualized by an imaging system (Bio-Rad, Hercules, CA, USA).

### Real-time PCR

Regional brain tissue including the ischemic core and penumbra was sectioned for real-time PCR analysis to examine the mRNA level of HIF-1α, and the corresponding region from the contralateral hemisphere was used as a control. RNA was extracted by TRIzol reagent (Invitrogen, Carlsbad, CA, USA). An amount of 400 ng RNA was used for the reverse transcription reaction with PrimeScript RT reagent kit (Takara, Dalian, China) according to the manufacturer’s instruction. The mRNA expression level was quantified with SYBR Premix Ex Tag Kit (Takara). The amplification parameters were 95 °C for 30 sec followed by 40 cycles of 95 °C for 5 sec and 60 °C for 30 sec. The measurement was conducted in triplicate. The expression level of HIF-1α mRNA was normalized to the level of reference gene β-actin and displayed as relative expression of mRNA by 2^−Δct^ method. HIF-1α forward primer was AAC TGC CAC CAC TGA TGA AT, reverse primer was CCA CTG TAT GCT GAT GCC TT. β-actin forward primer was CCT CTA TGC CAA CAC AGT, the reverse primer was AGC CAC CAA TCC ACA CAG.

### Statistical analysis

All results were expressed as mean ± SD. Data were analyzed by SPSS 18.0 for both parametric and nonparametric comparisons [49]. A probability value of *p* < 0.05 was considered as statistical significant.

## Results

### EPC isolation and characterization

**Fig. 1:**
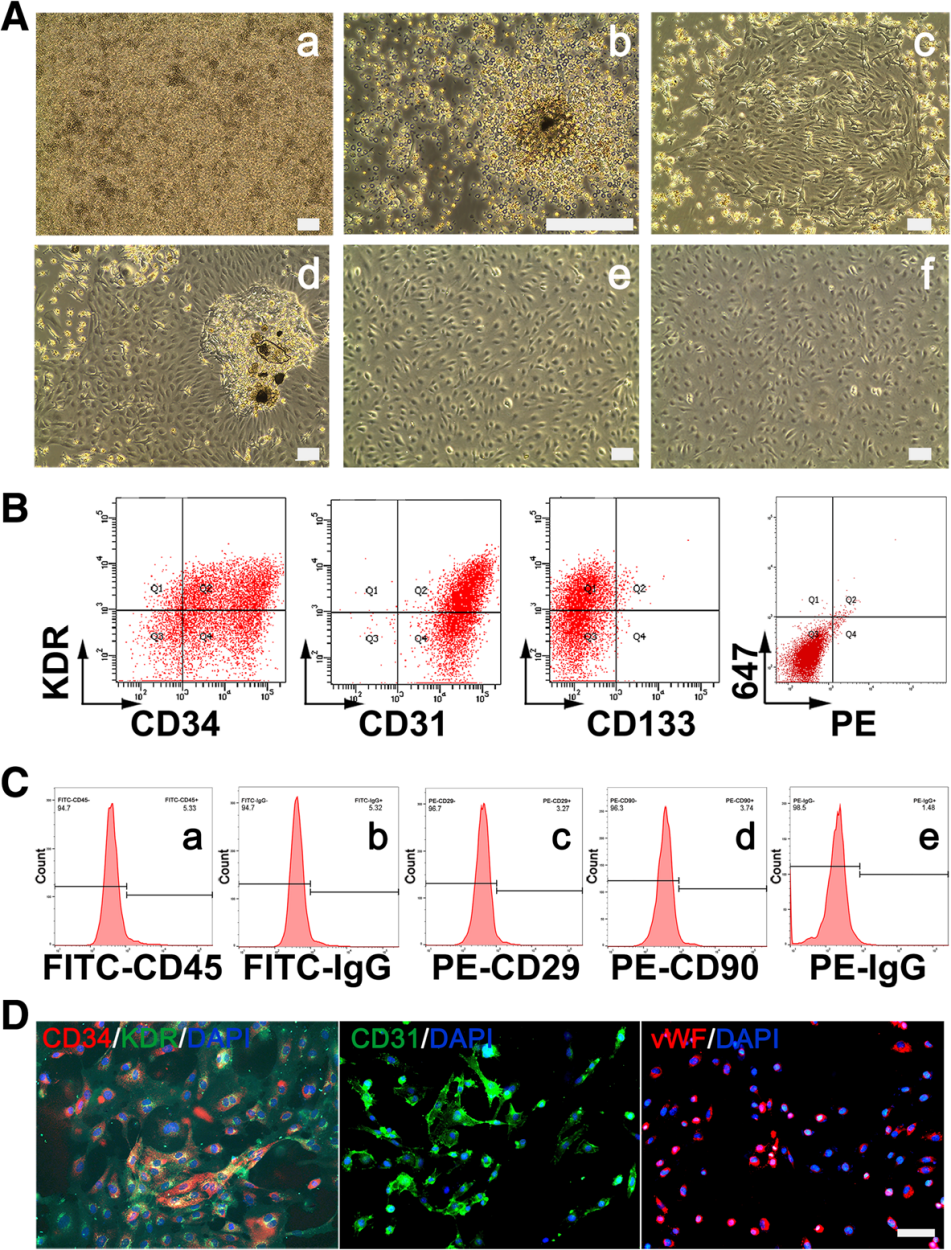
EPC isolation and identification. **A** Morphology of EPCs from initial seeding to passage 4 in cell culture. The cells showed different morphology throughout the time course. **a** Round monocytes with dispersal distribution were observed soon after seeding. **b** Early EPCs formed colonies at 7 days after seeding. **c** Expended EPCs formed colonies 21 days after seeding. **d** Expended EPCs were nearly confluent on day 28. **e** Passage 3 and (**f**) passage 4, EPC presented cobblestone phenotype. Scale bar = 50 μm. **B** Flow cytometric analysis showed the percentage of cultured EPC cells (about 50 days) with surface markers KDR^+^/CD34^+^, KDR^+^/CD31^+^, KDR^+^/CD133^+^ and isotype control (*from left to right*). **C** Flow cytometric analysis showed the percentage of culture EPC cells (about 50 days) with surface markers CD45^+^, CD 29^+^ and CD90^+^. **D** Cell immunofluorescent staining showed KDR^+^/CD34^+^, CD31^+^/vWF^+^ cells (about 50 days). Scale bar = 50 μm

EPCs from human umbilical cord blood were seeded in six-well plates coated with type I collagen. In the first week, the culture medium was partially replaced every 3 days in order to keep a small portion of non-adherent cells, which were needed to support the growth of expended EPCs by providing secreted cytokines. Early EPCs with a spindle-like morphology were observed to form colonies at 7 days after cell seeding; these cells gradually proliferated into expended EPCs with a cobblestone-like morphology at 14 days after culturing, and expanded rapidly into colonies at day 21. After 28 days of cell culture, larger colonies were observed and the culture became nearly confluent. Cells after passage 3 and 4 maintained cobblestone phenotype (Fig. 1A).

Cell surface markers CD34 and KDR are used for the identification of expended EPCs [51–53]. Flow cytometric analysis showed that CD34^+^/KDR^+^ cells accounted for 42.6%, and CD31^+^/KDR^+^ accounted for 55.3% of the total cell population, while CD133^+^/KDR^+^ cells were only 1.3% (Fig. 1B), which were consistent with reported characterizations of expanded EPCs. Flow cytometry showed that the cells were negative for CD90, CD45 and CD29 markers, indicating that these cells were not MSCs (Fig. 1C). Immunofluorescent staining showed that the most of KDR^+^ cells were co-stained with CD34, indicating that these cells were EPCs (Fig. 1D).

### EPC transplantation improved neurological outcome and decreased brain infarct volume

**Fig. 2:**
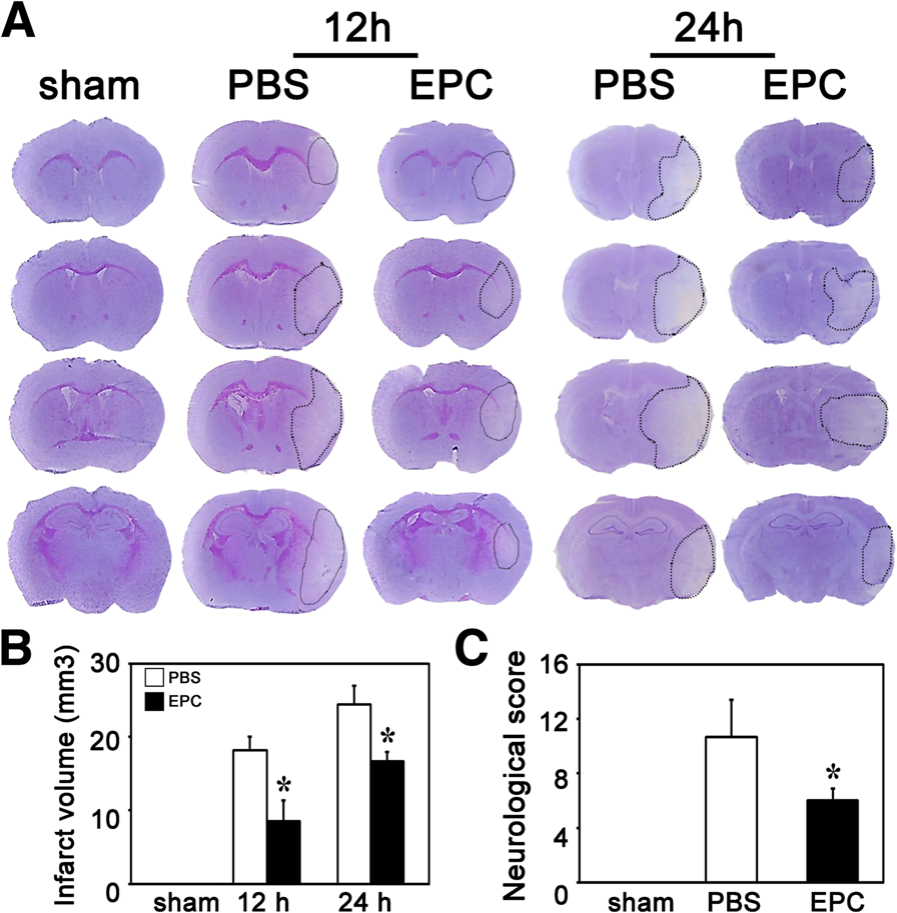
EPCs transplantation decreased brain infarct volume and attenuated neurological impairment after tMCAO in diabetic mice. **A** Representative photographs of brain coronal sections with Cresyl Violet staining of the sham, PBS-treated and EPC-treated diabetic mice after 12 h and 24 h of tMCAO. **B** Bar graph represents the quantification of the infarct volumes in the sham, PBS-treated and EPC-treated diabetic mice. Data are expressed as mean ± SD, n = 6–8 per group. ^*^ *p* < 0.05, EPC-treated vs. PBS-treated mice. **C** Bar graph shows the mNSS in the sham, PBS-treated and EPC-treated mice. Data are mean ± SD, n = 10–12 per group. ^*^ *p* < 0.05, EPC-treated vs. PBS-treated mice. *EPC* endothelial progenitor cell, *PBS* phosphate-buffered saline

To evaluate the effect of EPC transplantation on the brain injury after ischemia, Cresyl Violet staining was used to examine the brain infarct volume. We found that the infarct volume was significantly smaller at 12 h and 24 h after tMCAO in the EPC-treated diabetic mice compared to the PBS-treated diabetic mice (*p* < 0.05, Fig. 2A, B).

To explore the effect of EPCs transplantation on the neurological function after MCAO, we evaluated neurological behavior using mNSS scoring metrics at 24 h after tMCAO. We found that the neurological impairment in the EPCs-treated diabetic mice was attenuated compared to the PBS-treated mice (*p* < 0.05, Fig. 2C).

### EPC therapy attenuated the BBB impairment after tMCAO in diabetic mice

**Fig. 3:**
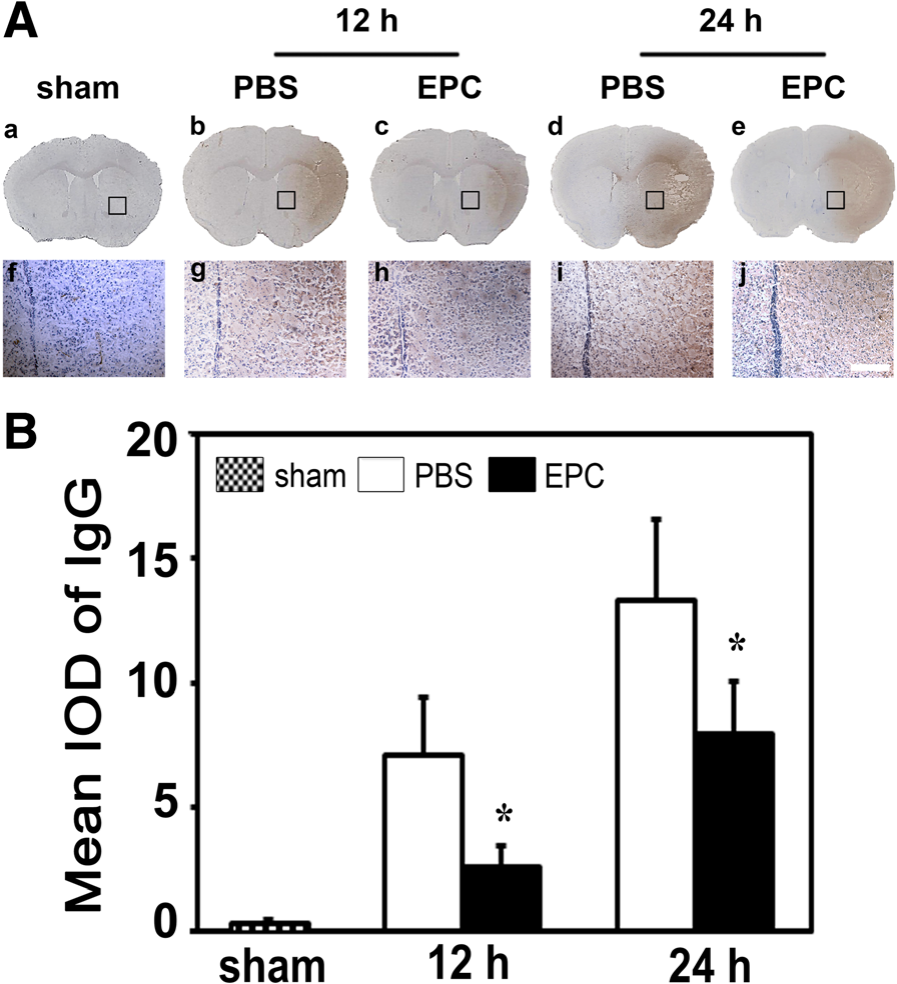
EPCs transplantation reduced the leakage of IgG after tMCAO in diabetic mice. **A** Photomicrographs shows the leaked IgG protein in *brown color* from brain vessel at 12 h and 24 h after tMCAO. Higher magnification of the boxed area in **a** to **e** were shown in **f** to **j**, respectively. Scale bar = 200 μm. **B** Bar graph shows the quantification of IgG protein in the sham, PBS-treated and EPC-treated mice. Data are mean ± SD, n = 6–8 per group. ^*^ *p* < 0.05, EPC-treated vs. PBS-treated mice. *EPC* endothelial progenitor cell, *IgG* immunoglobulin, *PBS* phosphate-buffered saline

**Fig. 4:**
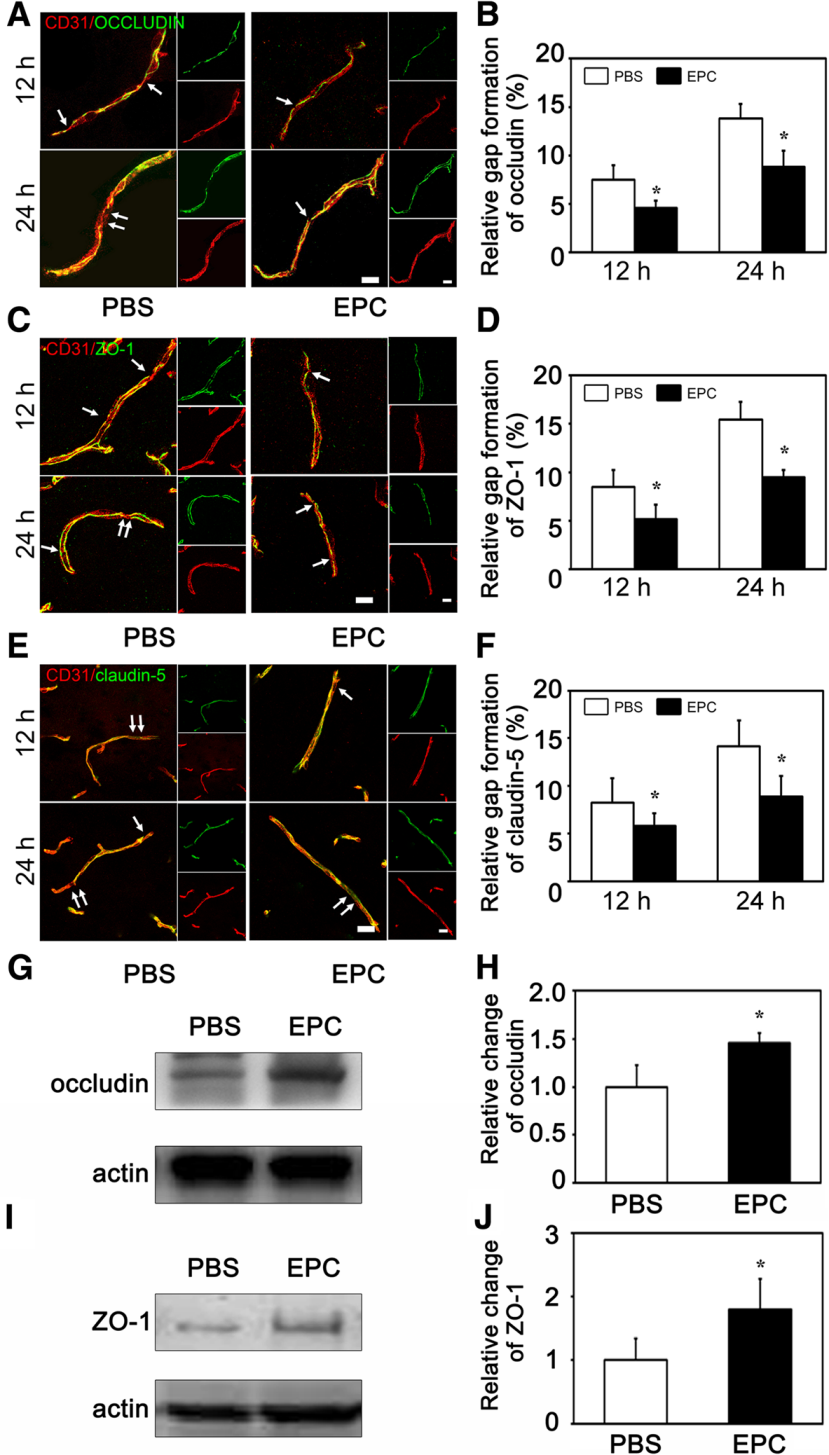
Gap formation of TJ in the EPC-treated and PBS-treated diabetic mice after tMCAO. **A** Representative image of TJ protein occludin (*green*) and endothelial marker CD31 (*red*) at12 h and 24 h after tMCAO in the PBS-treated and EPC-treated mice, scale bar = 10 μm. **B**. Bar graph shows the quantification of gap formation of occludin from figure (**A**). Data are mean ± SD, n = 6–8 per group. ^*^ *p* < 0.05, EPC-treated vs. PBS-treated mice. **C** Representative image of TJ protein ZO-1 (*green*) and endothelial marker CD31 (*red*), scale bar = 10 μm. **D**. Bar graph shows the quantification of gap formation of ZO-1 from figure (**C**). Data are mean ± SD, n = 6–8 per group. ^*^ *p* < 0.05, EPC-treated vs. PBS-treated mice. **E** Representative image of TJ protein claudin-5 (*green*) and endothelial marker CD31 (*red*), scale bar =10 μm. **F** Bar graph shows the quantification of gap formation of claudin-5 from figure (**E**). Data are mean ± SD, n = 6–8 per group. ^*^ *p* < 0.05, EPC-treated vs. PBS-treated mice. Western blot analysis shows the occludin (**G**) and ZO-1 expression (**I**) in the EPC-treated and PBS-treated mice at 24 h after tMCAO. **H** and **J** Bar graphs show the quantitative data of occludin and ZO-1 protein expression. Data are mean ± SD, n = 4 per group. ^*^ *p* < 0.05, EPC-treated vs. PBS-treated mice. *EPC* endothelial progenitor cell, *PBS* phosphate-buffered saline

To evaluate the BBB permeability in diabetic mice, we measured IgG leakage at 12 h and 24 h after tMCAO in diabetic mice. We demonstrated that IgG extravasation increased in the ischemic hemisphere after tMCAO while it was alleviated in the EPC-treated diabetic mice compared to the PBS-treated diabetic mice (*p* < 0.05, Fig. 3). CD31/occludin, CD31/ZO-1 and CD31/claudin-5 double staining results demonstrated that the continuous expression of TJ proteins on the endothelial cells in the normal brain while the presence TJ proteins became discontinuous and the gaps were enlarged in the tMCAO mice. The enlarged gaps were reduced in the EPC-treated mice compared to the PBS-treated diabetic mice. Semi-quantified Western blot results of occludin and ZO-1 paralleled with immunostaining results (*p* < 0.05, Fig. 4).

### PX-478 counteracted EPCs benefit and exacerbated brain injury after ischemia

**Fig. 5:**
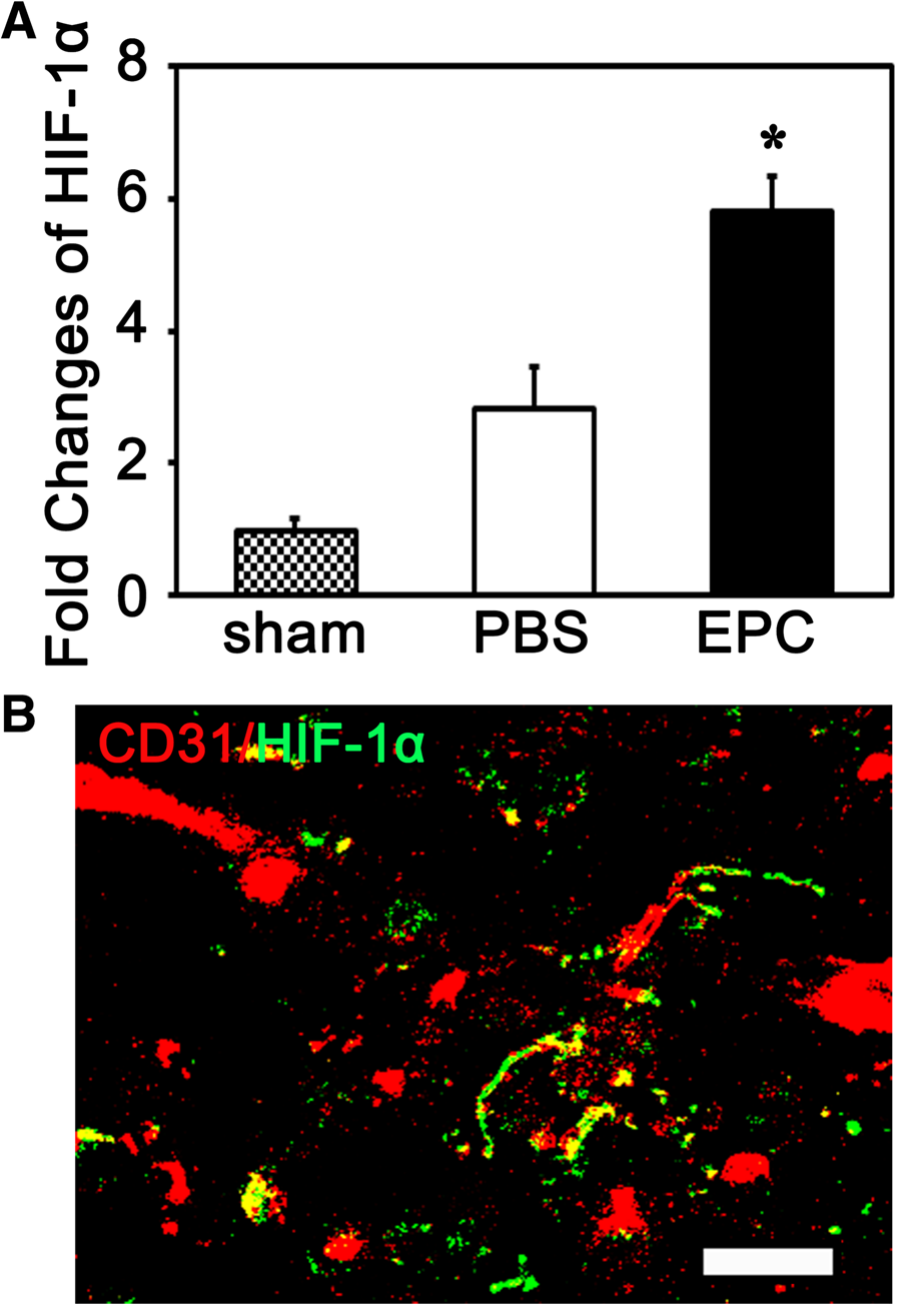
HIF-1α mRNA expression at 24 h after tMCAO in diabetic mice. **A** Bar graph shows the quantification of HIF-1α mRNA level in the sham, PBS-treated and EPC-treated brain tissue at 24 h after tMCAO. Data were normalized to the internal control. Data are as mean ± SD, n = 4 per group. ^*^ *p* < 0.05, EPC-treated vs. PBS-treated mice. **B** Photomicrograph showed that CD31 (*red color*) and HIF-1α (*green color*) double immunostaining. *Yellow color* indicated that CD31 and HIF-1α well merged, indicating ECs could express HIF-1α. Scale bar = 25 μm. *EPC* endothelial progenitor cell, *HIF-1α* hypoxia-inducible factor-1α, *PBS* phosphate-buffered saline

To explore the possible mechanism of EPC transplantation-mediated BBB protection after ischemic stroke in diabetic mice, we examined the level of HIF-1α in the ischemic brain of diabetic mice after EPC treatment. We found that the mRNA level of HIF-1α was upregulated after tMCAO, and it was further enhanced in the EPC-treated mice than that in the PBS-treated mice (*p* < 0.05, Fig. 5A). We also found colocalization of HIF-1α and CD31, indicating the endothelial cells could express HIF-1α (Fig. 5B).

**Fig. 6:**
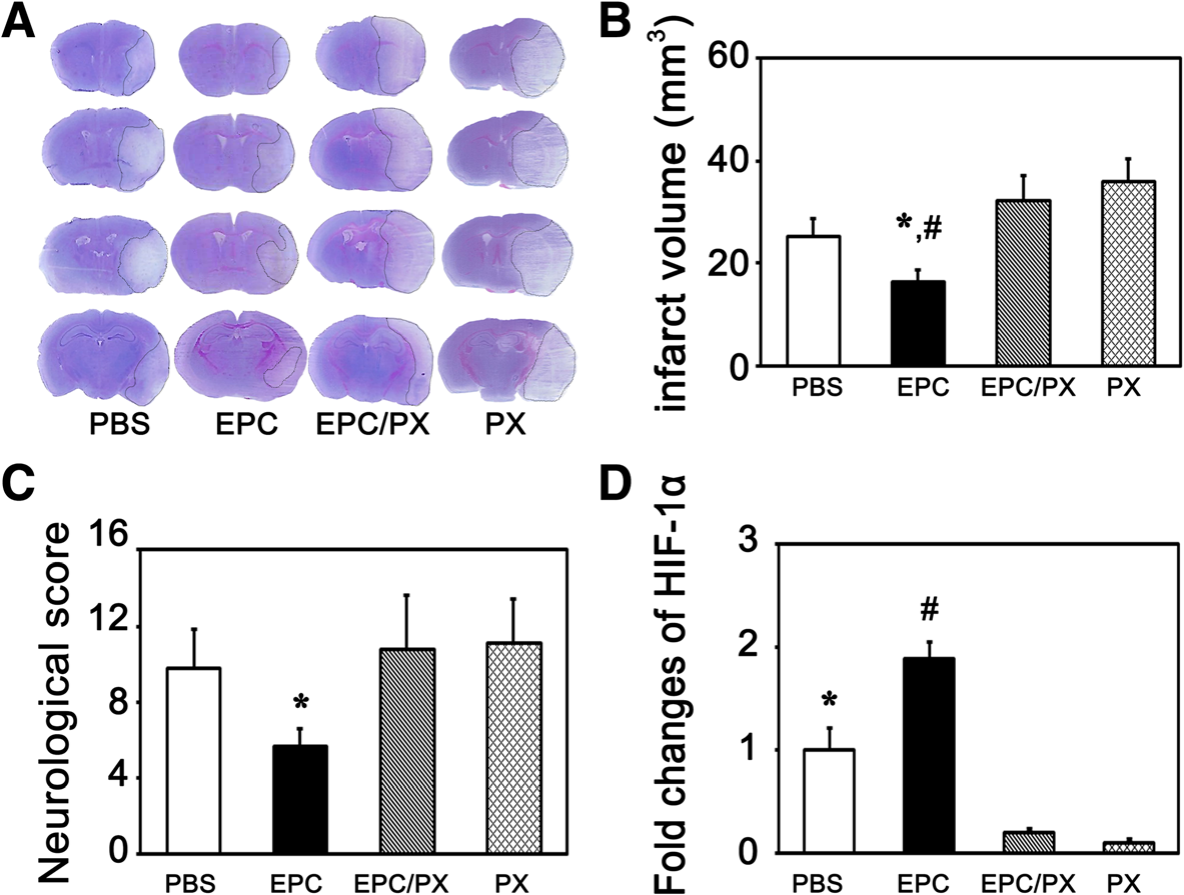
PX-478 increased brain infarct volume and exacerbated neurological deficit. **A** Photographs shows the brain infarct volume in the PBS-treated, EPC-treated, EPC and PX-478 co-treated and PX-478 alone mice at 24 h after tMCAO. **B** Bar graph shows the quantification of the infarct volume in the four aforementioned groups, respectively. Data are mean ± SD, n = 6–7 per group. *p < 0.05, EPC-treated vs. PBS-treated group. ^#^ *p* < 0.01, EPC- treated vs. EPC and PX-478 co-treated and PX-478 alone group. **C** Bar graph shows the mNSS in the EPC-treated, PBS-treated, EPC and PX-478 co-treated and PX-478 alone group at 24 h after tMCAO. Data are mean ± SD, n = 10–11 per group. ^*^ *p* < 0.05, EPC-treated vs. PBS-treated mice, EPC and PX-478 co-treated and PX-478 alone group. **D** Bar graphs shows HIF-1α mRNA level in the four aforementioned groups at 24 h after tMCAO. Data are mean ± SD, n = 4 per group. *p < 0.05, EPC-treated vs. PBS-treated group. ^#^ *p* < 0.01, EPC- treated vs. EPC and PX-478 co-treated and PX-478 alone group. PX = PX-478-treated mice. *EPC* endothelial progenitor cell, *HIF-1α* hypoxia-inducible factor-1α, *PBS* phosphate-buffered saline

**Fig. 7:**
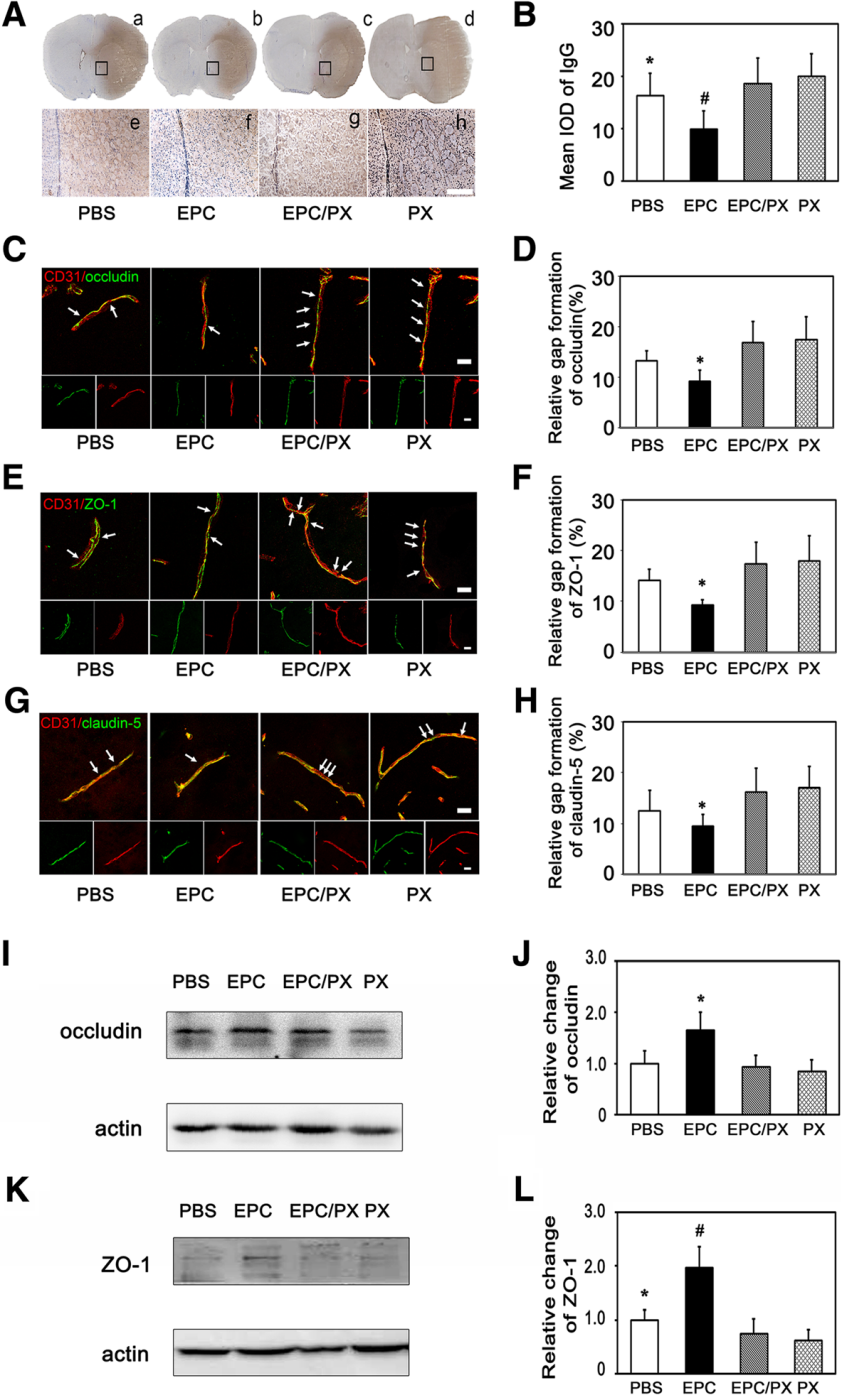
PX-478 increased BBB permeability after ischemia in diabetic mice. **A** Representative microphotographs of IgG-positive staining in the PBS-treated, EPC-treated, EPC and PX-478 co-treated and PX-478 alone group at 24 h after tMCAO. Higher magnification of the boxed area in **a** to **d** were shown in **e** to **h**. Scale bar = 200 μm. **B**. Bar graph shows the quantification of IgG-positive staining from figure **A**. Data are mean ± SD, n = 6–7 per group. ^*^ *p* < 0.05, EPC- treated vs. PBS-treated mice. ^#^ *p* < 0.01, EPC-treated vs. EPC and PX-478 co-treated and PX-478 alone group. **C**, **E**, **G**. Microphotographs showing the occludin/CD31, ZO-1/CD31 and claudin-5/CD31 immunofluorescence staining in the four aforementioned groups. *Arrows* indicated the location of reduced occludin, ZO-1 or claudin-5 staining, scale bar = 10 μm. **D**, **F**, **H**. Bar graphs showing the quantification of relative gap formation of occludin, ZO-1 and claudin-5 after 24 h of tMCAO. Data are mean ± SD, n = 6–7 per group. ^*^ *p* < 0.05, EPC-treated group vs. PBS-treated, EPC and PX-478 co-treated and PX-478 alone group. Western blot analysis showed the occludin (**I**) and ZO-1 expression (**K**) in the four groups. **J** and **L**. Bar graphs showed the quantitative data from figure **I** and **K**. Data are mean ± SD, n = 4 per group. ^*^ *p* < 0.05, EPC-treated vs. PBS-treated, EPC and PX-478 co-treated and PX-478 alone group in figure J. ^*^ *p* < 0.05, EPC-treated vs. PBS-treated group. ^#^ *p* < 0.01, EPC-treated vs. EPC and PX-478 co-treated and PX-478 alone group in figure L. PX = PX-478 treated mice. *EPC* endothelial progenitor cell, *IgG* immunoglobulin, *PBS* phosphate-buffered saline

To further verify that HIF-1α is involved in the beneficial role of EPCs in diabetic mice after ischemic stroke, HIF-1α inhibitor PX-478 was administrated after EPC injection. We found that EPC treatment decreased the brain infarct area after ischemia compared to the PBS-treated diabetic mice, while PX-478 blocked the beneficial effect of EPCs on reducing brain infarct volume and neurological deficiency in tMCAO mice (Fig. 6A-C). The mRNA level of HIF-1α was decreased after PX-478 treatment in the EPC-treated mice (Fig. 6D). PX-478 treatment also reversed the protective role of EPCs on the integrity of BBB (Fig. 7).

## Discussion

In the present study, we demonstrated that EPC transplantation attenuated infarct volume, reduced BBB leakage, and improved neurological outcomes following tMCAO in diabetic mice. Furthermore, we showed that HIF-1α inhibitor PX-478 could partially block the beneficial role of EPCs in protecting BBB integrity. These findings suggest that the beneficial effect of EPC therapy during ischemic stroke is associated with HIF-1α in diabetic mice.

The function of BBB is critical during ischemic stroke. BBB disruption could disturb microenvironments and cause severe edema, consequently induce neuronal apoptosis. Studies demonstrated that diabetes-induced microvascular disorder could lead to BBB disruption. Clinical studies found that BBB permeability increased in patients with diabetes in MRI [54, 55]. In vitro and in vivo studies showed that many factors were involved in the mechanism of BBB functional impairment in diabetes including the decrease of TJ protein [56, 57], the increase of MMP levels in blood plasma [58, 59], the participation of oxidative stress [60–62], and the elevation of inflammation response [63–65]. The BBB dysfunction in diabetes was widely considered responsible for the poor prognosis of ischemic stroke [4]. Moreover, the effect of certain treatment for ischemic stroke in non-diabetics could be different from that in the diabetics. Stem cell transplantation has become an attractive strategy for the treatment of ischemic stroke. MSCs were shown to improve the functional recovery after stroke in non-diabetic rats [9]. However, MSC therapy might increase the risk of BBB damage in diabetic rats [10]. The disturbance of BBB function in diabetes could increase the brain injury during ischemia, and this process was much more intricate and complicated, which led to the different treatment outcomes between diabetics and non-diabetics after ischemic stroke. Thus, the function of BBB was regarded as the key of ischemic stroke in patients with diabetes. The results from this study demonstrated that EPC treatment significantly reduced brain infarct volume and attenuated BBB leakage during the acute phase of tMCAO in diabetic mice, suggesting that EPC holds potential for stroke treatment, especially in diabetes.

In this study, un-labeled human EPCs were injected intravenously. Hence, we were unable to track the distribution of injected EPCs after transplantation. We previously reported that some of the intravenously transplanted EPCs can home to the ischemic perifocal region and improve long-term neurobehavioral outcomes after tMCAO [48]. Our previous work also suggested that intravenously transplanted stem cells mainly homed to the lung, while imparted neuroprotective effect via secreting neuroprotective factors [66]. Our current data supports that the transplantation of EPCs protect BBB function, most possibly indirectly through regulation of HIF-1α and its downstream effectors in diabetic mice.

HIF-1α is considered as the master transcriptional regulator in response to changes in oxygen levels [67, 68]. Although the experimental evidence from in vitro and in vivo studies showed that the level of HIF-1α is significantly upregulated in the focal ischemic brain [43, 69, 70], the role of HIF-1α in the pathophysiology of ischemic stroke is controversial. Many studies supported the neuroprotective effect of HIF-1α in ischemic models [43–46], with the mechanisms involving the production of erythropoietin [71], anti-apoptosis [72], and suppression of p53 activation, etc. [73]. Others demonstrated that the increase of HIF-1α expression under hypoxic conditions induced apoptosis [74], promoted hypoxia-induced delayed neuronal death [75], and enhanced the brain damage induced by ischemia [69, 76]. Studies also showed that HIF-1α inhibitor had a protective effect on the BBB permeability [77, 78] while it did not have the same effect on the edema formation [79]. Our results showed that the mRNA level of HIF-1α increased in the ischemic brain as well as the EPC-treated diabetic mouse brain. IgG protein leakage was reduced by EPC treatment but exacerbated in the EPC and PX-478 co-treated mice. Furthermore, the gap formation of brain microvascular endothelial cells was enlarged in the PX-478-treated diabetic mice. These results suggested that the inhibition of HIF-1α compromised BBB integrity and increased BBB permeability. Upregulated HIF-1α expression is associated with the protective effect of EPC treatment on BBB integrity in the diabetic mouse brain following tMCAO. However, current data cannot delineate whether HIF-1α regulates TJ protein expression directly or through other mediators. Brain endothelial cells, pericytes, neuron and astrocytes all express HIF-1α. Endothelial cells exhibited greater response to ischemic injury in the upregulation of HIF-1α expression compared to astrocytes and pericytes [80–83]. Other studies supported that brain endothelial cell HIF-1α can directly regulate the expression of occludin and ZO-1, albeit with controversial outcome [84–86]. The differential effect of HIF-1α on BBB integrity observed in different studies could result from differences in experimental conditions including animal species, different ischemic animal models and methods of measurement. The mechanism by which HIF-1α affects BBB integrity and permeability in acute ischemic stroke warrants further study.

## Conclusions

Our work demonstrated that EPC therapy significantly attenuated the BBB disruption in the acute phase of ischemic stroke in diabetic mice, which consequently alleviated neurological deficits and reduced cerebral infarct volume. HIF-1α played an important role in the protective effect of EPCs.

## Abbreviations

BBB: Blood-brain barrier
EPC: Endothelial progenitor cell
HIF-1α: Hypoxia-inducible factor-1α
IgG: Immunoglobulin G
MCAO: Middle cerebral artery occlusion
mNSS: Modified neurological severity score
MSC: Mesenchymal stem cell
PBS: phosphate-buffered saline
STZ: Streptozotocin
TJ: Tight junction
